# Development and validation of nomogram for predicting survival of elderly patients with stage I small-cell lung cancer

**DOI:** 10.17305/bjbms.2020.5420

**Published:** 2021-10

**Authors:** Yaji Yang, Shusen Sun, Yuwei Wang, Feng Xiong, Yin Xiao, Jing Huang

**Affiliations:** 1 Department of Anesthesiology, Chongqing Medical University, Chongqing, China; 2 Department of Pharmacy Practice, College of Pharmacy and Health Sciences, Western New England University, Springfield, United States; 3 Department of Pharmacy, Xiangya Hospital Central South University, Changsha, Hunan, China; 4 Department of Radiotherapy, Chongqing University Cancer Hospital, Chongqing, China; 5 Department of Respiratory and Critical Care Medicine, The First Affiliated Hospital of Chongqing Medical University

**Keywords:** Nomogram, small-cell lung cancer, SEER, stage I, elderly

## Abstract

There is a lack of predictive models to determine the prognosis of elderly patients diagnosed with Stage I small-cell lung cancer (SCLC). The purpose of this study was to establish a useful nomogram to predict cancer-specific survival (CSS) in the elderly patient population. Based on the Surveillance, Epidemiology, and End Results registry database, patients aged ≥ 65 years with pathological AJCC (American Joint Committee on Cancer) Stage I SCLC from 2004 to 2014 were identified. The CSS was evaluated by the Kaplan-Meier method. Patients were randomly split into training and validation sets. In the training cohort, univariate analysis and multivariate analysis using the Cox regression identified risk factors that affected CSS, and the results were utilized to construct a nomogram for prediction of the 1-, 3-, and 5-year CSS rates of elderly patients with Stage I SCLC. The effectiveness of the nomogram was validated internally and externally by the bootstrap method. The clinical practicability and accuracy of the nomogram were evaluated by the concordance index (C-index), calibration curve, receiver operating characteristic curve, and decision curve analysis. In total, we extracted 1,623 elderly patients with Stage I SCLC. The median CSS was 34 months, and the 5-year CSS was 41%. Multivariate analysis revealed that histologic type, tumor size, age, and AJCC Stage were significant predictors of CSS. A nomogram was constructed according to the results of multivariate COX analysis. The C-indices of the nomogram for training and validation sets were 0.68 and 0.62, indicating that the nomogram demonstrated a favorable level of discrimination. The calibration curves exhibited satisfactory agreement between the actual observation and nomogram prediction. The net benefit of the nomogram was better than the AJCC TNM staging. A practical nomogram to predict the CSS of elderly patients with Stage I SCLC is constructed. The predictive tool is helpful for patient counseling and treatment decision-making.

## INTRODUCTION

Lung cancer is one of the most common cancers and the leading cause of cancer mortality globally. [1] Small-cell lung cancer (SCLC) is the most aggressive type of lung cancer characterized by paraneoplastic endocrinopathy, high cellular proliferation, and early metastatic spread. [2] With an aging population, the number of elderly individuals with lung cancer is expected to increase in the near future. Additionally, a growing number of patients are diagnosed at an earlier stage with widespread low-dose and thin-slice computed tomography screening. [3]

The National Comprehensive Cancer Network (NCCN) Guidelines indicate that for Stage I SCLC patients without mediastinal lymph node metastasis lobectomy with mediastinal lymph node dissection followed by systemic therapy should be considered to offer a potential cure in clinical practice. [4,5] Moreover, due to the high heterogeneity and poor differentiation within SCLC, non-surgically-resected samples might fail to identify SCLC correctly. Surgical resection is regarded as a more precise approach to get a considerable number of specimens for the diagnosis [6]. An increasing number of studies revealed a promising prognosis in patients with early-stage SCLC undergoing resection [7-9].

However, surgery is associated with a high risk of cancer recurrence and postoperative morbidity. Postoperative complications after surgery for SCLC are accompanied by higher short-term mortality and reduced long-term survival. [10] A previous study indicated that elderly patients with lung cancer following surgery have much higher post-operative complications (26.0% vs. 13.3%) and mortality rate(8.2% vs. 2.2%) than younger patients. [11] Relapse rates among patients following surgical resection are as high as 21-56%. [12, 13] Thus, appropriate surgery candidate selection would contribute to a higher quality of life and improved morbidity, both of great clinical significance. It is useful to build a clinically applicable and simple scoring system to select patients with a better prognosis undergoing surgical procedures. Substantial heterogeneity exists among SCLC patients in demographic and clinicopathological features, such as age, gender, pathological type, and tumor grade. The prognosis of SCLC varied among different individuals. It is essential to stratify stage I SCLC into diverse prognostic categories to distinguish patients who can most benefit from postoperative adjuvant therapies.

Nomograms, which create a visual manifestation of a statistical predictive model outputting a numerical probability of a clinical outcome, are commonly used to evaluate prognosis in cancer patients. [14] Nomograms guide clinical decision-making and add value for risk stratification, personalized treatment, and clinical trial design. A recent study has established a nomogram to predict the mortality of stage I SCLC patients. [15] The nomogram used data on patient characteristics and treatments extracted from the Surveillance, Epidemiology, and End Results (SEER) database. The study included three age groups, 18.9% of the patients aged < 60 years, 54.2% aged 60–75 years, and 26.9% aged > 75 years. However, the nomogram had not undergone external validation, and a predictive model specifically for elderly patients diagnosed with stage I SCLC still needs to be constructed.

In the current study, we attempted to build and validate a nomogram to identify risk factors affecting cancer-specific survival (CSS) in elderly patients with Stage I SCLC based on data derived from the SEER database.

## MATERIALS AND METHODS

### Data source

All patient data were extracted from the National Cancer Institute NCI SEER database using the SEER*stat software (version 8.3.5; http://seer.cancer.gov/seerstat/). The SEER database is sponsored by the NCI to collect information about cancer incidence and outcome. The database is updated annually and includes information on survival and follow-up. [16]

### Patient selection and data collection

Tumor histology and site are coded in SEER according to the International Classification of Diseases (ICD) for Oncology Edition 3 (ICD-O-3). [17] Patients with ICD-O-3 histology code 8041/3–8045/3 of the lung and ICD-O-3 site code c34.0-c34.9 treated from 2004 to 2014 were identified. Demographic and clinicopathologic variables were documented for all patients studied. The variables included sex, age at diagnosis, race, laterality, primary site, tumor size, histologic types, the American Joint Committee on Cancer (AJCC) TNM stage (6th edition), survival data, and vital status. Because we narrowed our focus to surgery candidates first diagnosed with SCLC, not all treatment data were included in the study.

The patient inclusion criteria were (a) diagnosed as early-stage (AJCC stage I) SCLC; (b) at least 65 years of age at diagnosis; and (c) histologically confirmed malignancy. Exclusion criteria were (a) patients with missing data, including unknown age of diagnosis; uncertain race; unknown tumor size; unknown cause of death; (b) survival was unknown or less than one month after diagnosis; and (c) patients with more than one primary cancer during follow-up.

### Endpoint definition

SCLC Cancer-specific death was defined as “patient death from SCLC as the underlying cause” in SEER. The endpoint of the present study was CSS, the period between the SCLC diagnosis and death due to cancer-specific death, with deaths from other causes censored. Follow-up was concluded on 31 December 2014.

### Construction and validation of the nomogram

The included patients were divided into the training and validation cohorts randomly. The training cohort was applied to establish the nomogram. The Kaplan-Meier method was utilized to evaluate survival curves, and they were compared using the log-rank test. Variables with a p-value < 0.05 in univariate analysis were included in the multivariate analysis. The multivariate analysis was performed with Cox proportional hazards regression model. The nomogram was constructed with potential risk factors (p < 0.05) based on the multivariate Cox analysis.

The nomogram was subjected to bootstrap resampling 1,000 times for internal validation in the training set and external validation in the validation cohort. The nomogram’s effectiveness was assessed by the concordance index (C-index), calibration plots, and receiver operating characteristic (ROC) curve. The C-index (ranges 0.5 to 1) was used to measure discrimination. A higher C-index value indicates a more accurate prognostic prediction. [18] The calibration plots of the nomogram were used to assess the consistency between the actual and predicted survival outcomes. Decision curve analysis (DCA) was performed to evaluate the nomogram’s clinical net benefit.

### Ethical statement

Our study did not require an ethical board approval because it did not contain human or animal trials.

### Statistical analysis

Continuous variables were presented as means ± SD. Frequencies and proportions were calculated for categorical variables. To compare the characteristics of patients between the training and validation cohorts, a Student’s t-test was used for continuous variables and a Pearson Chi-Squared test for categorical variables. Statistical analysis was performed using SPSS 24.0 software. The package of rms in R software was used to construct the nomogram. [19]

## RESULTS

A total of 1,623 patients from the SEER database were identified and included in the study (Table 1). The median age was 74 years (ranged from 65 to 96), and 790 patients (48.7%) were men. The majority, 1,444 (89.0%), were Caucasian. The upper lobe was the most common SCLC site with 917 patients (56.5%), followed by the lower lobe with 494 patients (30.4%). The most frequent histologic type was “Small cell carcinoma, not otherwise specified (NOS).” The AJCC stage included 53.9% IA and 46.1% grade IB.

**TABLE 1:**
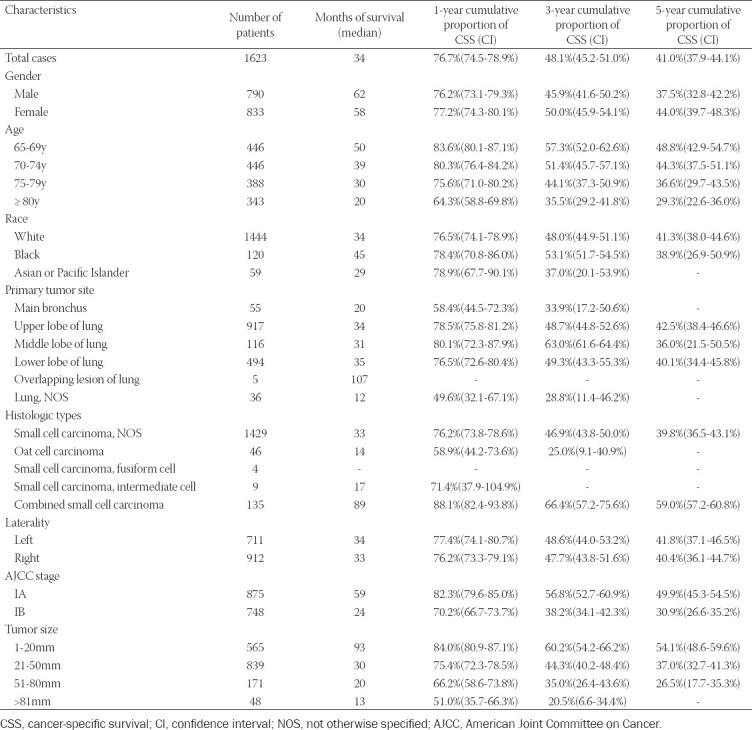
Patient characteristics and 1-, 3-, and 5-year lung cancer-specific survival

### Survival analysis

**FIGURE 1:**
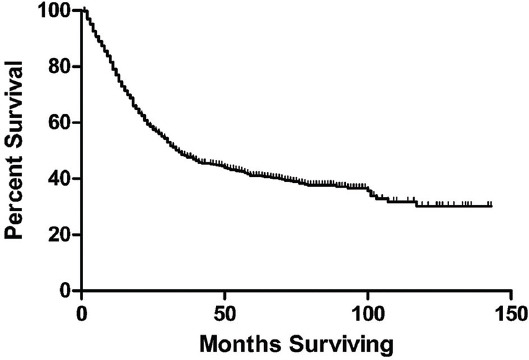
Kaplan-Meier survival curve of all included patients.

The CSS curve is shown in Figure 1. The median CSS was 34 months (range, 1–143 months). The 1-, 3-, and 5-year CSS rates were 76.7% (95% CI, 74.5% -78.9%), 48.1% (95% CI, 45.2% - 51.0%) and 41.0% (95% CI, 37.9% - 44.1%) respec¬tively (Table 1).

### Independent prognostic factors and nomogram development

**FIGURE 2:**
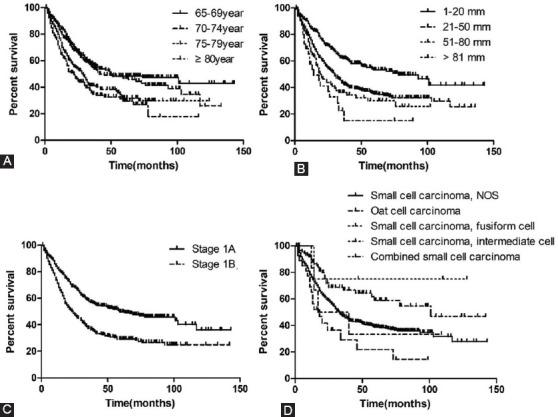
Kaplan-Meier survival curves for patients according to (A) Age, (B) Tumor size, (C) AJCC stage, (D) Histologic types

The clinicopathologic characteristics of lung cancer patients, the training set (n = 1,082) and validation set (n = 541), are listed in Table 2, respectively. In the training set, the univariate analysis demonstrated that gender, age at diagnosis, primary site, histologic type, AJCC stage, and tumor size was associated with CSS (Table 3). All significant risk factors in the univariate analysis were included for the multivariate analysis. The results of the Cox hazards regression analyses are shown in Table 3. Analysis indicated that age at diagnosis, histologic types, AJCC stage, and tumor size remained significant independent risk factors in the Cox regression model. The Kaplan-Meier survival curve for significant risk factors is presented in Figure 2.

**TABLE 2:**
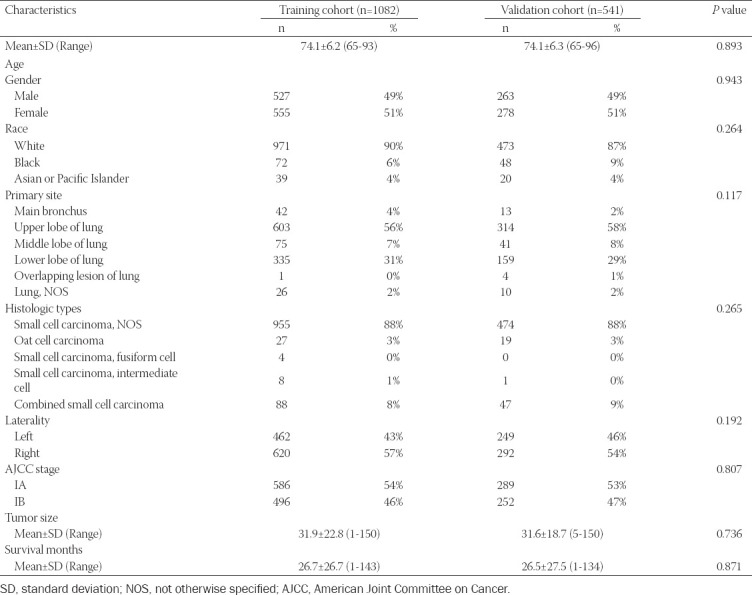
Characteristics of the training and validation cohorts

**TABLE 3:**
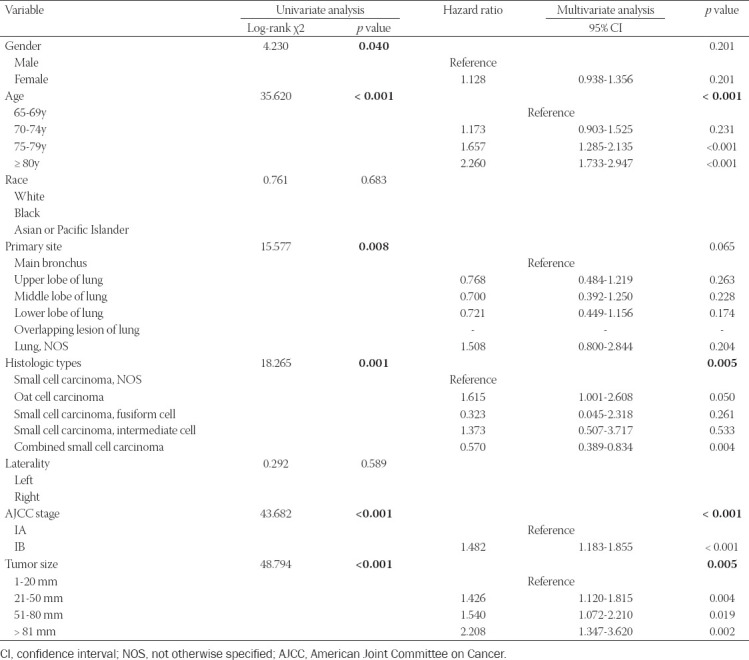
Univariate and multivariate Cox proportional hazards regression analyses for elderly patients with Stage I Small Cell Lung Cancer

**FIGURE 3:**
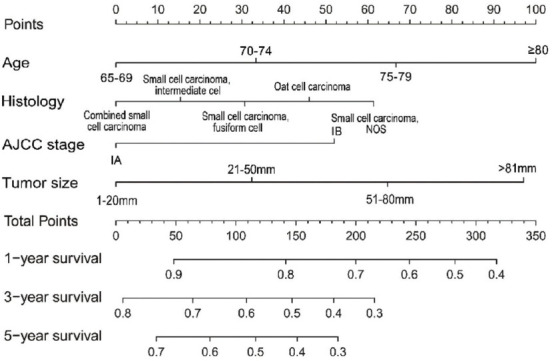
A nomogram for prediction of 1-, 3- and 5-year lung cancer-specific rates in elderly patients with Stage I Small Cell Lung cancer.

A nomogram containing the variables independently associated with CSS is presented in Figure 3. The CSS rates of 1-, 3-, or 5-year can be calculated by adding together the points corresponding to the patient’s characteristics.

### Validation of the nomogram

**FIGURE 4:**
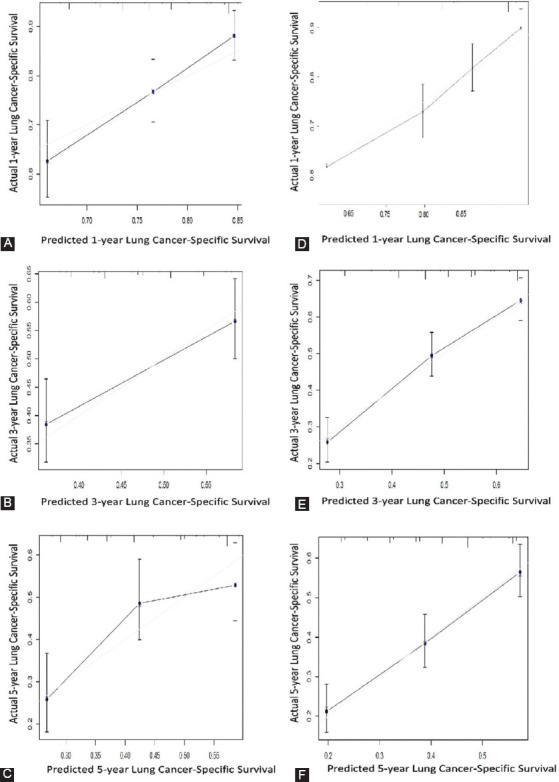
The calibration curves of 1-, 3- and 5-year lung cancer-specific survival rates of elderly patients with Stage I Small Cell Lung Cancer for training cohort (A, B, C) and for validation cohort (D, E, F).

Internal validation of the nomogram was conducted by using internal bootstrap analyses with 1,000 resamplings. The C-index of the prognostic nomogram for CSS prediction was 0.68. Similarly, external validation revealed that the C-index value for predicting CSS was 0.62. These findings reflected the good discrimination ability of the model in both the training and validation cohorts. The internal and external calibration curves are shown in Figure 4. A diagonal gray line represents the actual CSS probability, and a solid black line represents the model’s performance in forecasting the CSS probability. The two lines overlap closely, suggesting that the nomogram has reasonable estimations in both patient cohorts.

**FIGURE 5:**
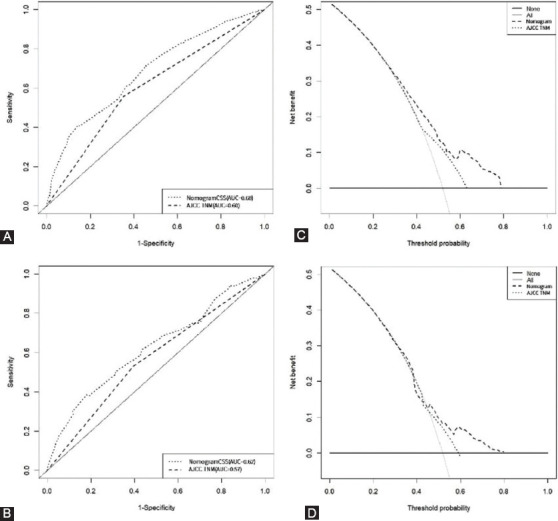
Receiver operating characteristic (ROC) curves and decision curve analysis (DCA) of the nomogram and AJCC TNM stage. (A) ROC for cancer-specific survival(CSS) in training set, (B) ROC for CSS in validation set, (C) DCA for CSS in training set, (D) DCA for CSS in validation set.

ROC graphically showed nomograms possessed better predictive performance compared with AJCC-staging. Furthermore, the areas under the curve (AUC) of the nomogram for CSS prediction in ROC analysis were 0.68 (95%CI: 0.64-0.73) and 0.62 (95%CI: 0.57-0.68) for training and validation sets, respectively. DCA results revealed that nomograms achieved a better net benefit than AJCC TNM staging, suggesting the clinical usefulness of the nomogram to predict CSS (Figure 5).

## DISCUSSION

This study established and validated a nomogram based on 1,623 elderly Stage I SCLC patients from the SEER database. The nomogram was used to predict the 1-, 3- and 5-year CSS rates of elderly patients with early-stage SCLC according to four significant factors: age at diagnosis, histologic type, AJCC stage, and tumor size. The nomogram is a reliable and straightforward predictive tool to estimate prognosis and make appropriate therapy recommendations.

The aging population is increasingly affected by lung cancer. Efforts should be made to optimize treatments for aging patients. The current model contains only the essential clinically signifiant variables. These variables are cheaper than molecular tests and provide a more practical option. The C-index implied that the model is satisfactory, and the internal calibration curves show good consistency between the actual observation and nomogram prediction. Hence, the reliability and repeatability of the nomogram could be ensured. [20] The predictive ability of our nomogram is consistent compared to the previous nomogram study for stage I SCLC (C-index, 0.68 vs. C-index, 0.69). [15] The nomogram could be potentially useful tool to identify high-risk patients, achieve more precise patient survival predictions, and optimize treatment.

A total of 30% of SCLC are defined as a limited-stage disease (characterized as disease confined to one hemithorax that can be compassed in a tolerable radiation field) or stage I-III based on the TNM classification. [21] Compared to extensive-stage disease (defined as disease beyond that included in the limited-stage disease), the 5-year survival rate of limited-stage I SCLC is much higher (1.6% vs. 12.1%). [22,23] Surgery plays a crucial role in limited-stage SCLC. Combs et al. indicated that the 5-year overall survival (OS) for stage I of resectable SCLC patients treated with surgery and chemotherapy reached 49%. [9] Both the ACCP and NCCN recommend surgery with adjuvant chemotherapy for stage I disease. [24,25] Li and his colleagues recently constructed a nomogram for predicting CSS in patients diagnosed with stage I SCLC. Their results show that surgery, chemotherapy, and radiotherapy could improve the one-year survival rate. Surgery effectively reduces cancer-specific mortality, with the one-year cumulative incidence drops from 34.5% to 11.2%. [15]

Overall, in clinical practice, the use of surgery and optimal treatment with chemotherapy in the elderly population remains low, leading to under-treatment and undesirable survival rates. [26,27] Most elderly stage I SCLC patients are concerned about undergoing surgery, chemotherapy, and thoracic radiation aware of the risk and side effects of these treatment options. Therefore, there is an urgent need to build a scoring system to guide patients. Besides, our study was aimed to assess the prognosis at the onset of diagnosis in the elderly SCLC patients. The nomogram helps select appropriate candidates for surgery, optimize the allocation of medical resources, and establish effective communication between patients and physicians.

By performing a comparison among four age cohorts (65-70 years, 70-75 years, 75-80 years, and > 80 years), we found that the 5-year CSS of the >80-years group was the lowest (29.3% compared with 36.6%, 44.3%, 48.8%). Similar to previous studies, the current analysis confirms that age is an independent prognostic factor. [28-30] Similarly, a study of 1,303 patients enrolled in 11 trials show that older subjects, compared with younger subjects have worse progression-free survival (PFS), overall survival, comorbidity score, poorer performance, and difficulty in tolerating and completing therapy. All these factors probably contribute to lower survival. [31] Older age is directly linked with an unfavorable prognosis, and this variable is recommended to be considered in selecting treatment strategies. [32]

The different histological types of SCLC are significantly associated with biological characteristics and prognosis. As shown in the nomogram, the histological type is an important predictive factor of CSS. From the data on the cumulative incidence of death, the combined SCLC has a smaller percentage of mortality than other types of SCLC. Since 1981, the World Health Organization divided SCLC into three subtypes: oat cell, intermediate cell, and combined cell. Combined cell indicates combinations of malignant squamous and glandular elements. [33] Several retrospective studies have reported that combined SCLC has notable characteristic clinical features, frequently presenting with early-stage disease and potentially curable. This explains the possible benefit of multimodality therapies, including surgery, in patients with combined SCLC. [34,35]

Growing studies reveal that tumor size is an independent prognostic factor of survival in many cancers, including SCLC. [36-38] According to our research, tumor size significantly affects the survival rate. In patients with tumor size > 2 cm, CSS is substantially higher than in patients with a tumor size ≤ 2cm. Published research shows that tumor size is positively associated with the deficiency of a patient’s immune ability. [39,40] Therefore, the positive relationship between immunity deficiency and tumor size might be why tumor size is a prognostic factor in SCLC.

Our study has the following limitations: 1) potential inevitable bias due to the nature of retrospective data collection, [41] 2) the SEER program lacks data on pathologic characteristics , therapy and comorbidity, which may influence the prognosis, [42] 3) the predictive nomogram was constructed according to data derived from the SEER database which does not reflect the global population. Further, multi-center, large-sample studies are necessary to externally validate the nomogram to verify whether these results are generally applicable. Despite these limitations, the nomogram is constructed based on enormous population data collected in the SEER database leading to unique opportunities to forecast CSS for patients with Stage I SCLC.

## CONCLUSION

Nomogram is constructed to estimate the probability of CSS of elderly patients with Stage I SCLC based on cohorts from the SEER database. Model validation proves its satisfactory performance, being optimally accurate in predicting the prognosis of elderly patients with Stage I SCLC. The nomogram helps clinicians select individuals who can benefit the most from surgery, thus providing more individualized treatment strategies.
